# CD13-Mediated Pegylated Carboxymethyl Chitosan-Capped Mesoporous Silica Nanoparticles for Enhancing the Therapeutic Efficacy of Hepatocellular Carcinoma

**DOI:** 10.3390/pharmaceutics15020426

**Published:** 2023-01-28

**Authors:** Jinhu Liu, Weiwei Mu, Tong Gao, Yuxiao Fang, Na Zhang, Yongjun Liu

**Affiliations:** Department of Pharmaceutics, Key Laboratory of Chemical Biology (Ministry of Education), NMPA Key Laboratory for Technology Research and Evaluation of Drug Products, School of Pharmaceutical Sciences, Cheeloo College of Medicine, Shandong University, Jinan 250012, China

**Keywords:** mesoporous silica nanoparticle, NGR peptide, pegylated carboxymethyl chitosan, triggered release, hepatocellular carcinoma

## Abstract

Liver cancer, especially hepatocellular carcinoma, is an important cause of cancer-related death, and its incidence is increasing worldwide. Nano drug delivery systems have shown great promise in the treatment of cancers. In order to improve their therapeutic efficacy, it is very important to realize the high accumulation and effective release of drugs at the tumor site. In this manuscript, using doxorubicin (DOX) as a model drug, CD13-targeted mesoporous silica nanoparticles coated with NGR-peptide-modified pegylated carboxymethyl chitosan were constructed (DOX/MSN-CPN). DOX/MSN-CPN comprises a spherical shape with an obvious capping structure and a particle size of 125.01 ± 1.52 nm. With a decrease in pH, DOX/MSN-CPN showed responsive desorption from DOX/MSN-CPN and pH-responsive release of DOX was observed. Meanwhile, DOX/MSN-CPN could be efficiently absorbed through NGR-mediated internalization in vitro and could efficiently deliver DOX to tumor tissues with long accumulation times in vivo, suggesting good active targeting properties. Moreover, significant tumor inhibition has been observed in antitumor studies in vivo. This study provides a strategy of utilizing DOX/MSN-CPN as a nano-platform for drug delivery, which has superb therapeutic efficacy and safety for the treatment of hepatocellular carcinoma both in vivo and in vitro.

## 1. Introduction

Cancer ranks as a leading cause of death in most countries of the world. Among them, liver cancer is the third leading cause of cancer-related death, and its incidence is increasing worldwide [1]. Hepatocellular carcinoma is the dominant form of liver cancer. With the rapid development of nanotechnology, nano drug delivery systems provide a promising strategy for enhancing the efficacy of antineoplastic drugs [2]. These nanomedicines have multiple functions, such as prolonging the blood circulation time of drugs, enhancing the accumulation of drugs in tumor sites, promoting the uptake of tumor cells, controlling drug release, and so on [3]. Currently, several formulations, such as Doxil^®^ (doxorubicin, DOX), Abraxane^®^ (paclitaxel), and Lipusu^®^ (paclitaxel), are approved by the FDA for clinical cancer therapy [4]. After administration of the same dosage, nanomedicines exhibit significantly increased concentrations in tumor tissues in comparison to traditional solutions with fewer side effects. However, in one study, the therapeutic outcomes did not improve as expected and high drug dosages are still required in clinical therapy to increase antitumor efficiency [5]. In order to improve the therapeutic efficacy of hepatocellular carcinoma, it is very important to realize the high accumulation and effective release of drugs at the tumor site [6].

Active targeting nano drug delivery systems mainly use nanocarriers modified by targeting factors such as ligands and antibodies to deliver drugs to the target sites. Targeting factors on the surface of nanoparticles can increase the binding affinity of nanoparticles to cell membrane receptors, promoting receptor mediated endocytosis, thereby improving the accumulation of drugs in the lesion site [7]. The ability of active targeting depends critically on the proximity of the nanoparticles to the target antigen. In some cases, off-target effects, undesirable immunogenicity, and low circulation times reduce the availability of the nanoparticles and negatively affect their biodistribution [8]. To escape this dilemma, the discovery and application of specific targeting sites is vital. The asparagine–glycine–arginine (NGR) peptide is a promising targeting ligand which can recognize a tumor-specific isoform of aminopeptidase N (APN or CD13) [9]. It has been demonstrated that CD13 is overexpressed both in the tumor vasculature and tumor cell membranes [10,11]. Thus, the NGR peptide serves as a promising ligand targeting both tumor cells and tumor vasculature endothelial cells for a one-double targeting tumor therapy strategy. CD13-mediated nanoparticles have been designed for imaging and photodynamic therapy for tumors [12,13]. However, for antitumor drug stimulation response nano drug delivery systems, such studies are not yet enough.

Stimulus-responsive nano drug delivery systems can be constructed using nanocarriers modified with environmentally sensitive molecules. Due to the differences between tumor tissues and normal tissues in their signal characteristics such as their pH, specific enzyme concentration, or redox gradient, various stimulus-responsive nanocarriers are being developed for targeted delivery of drugs to tumor tissues and cells. Among them, the construction of pH-sensitive nanocarriers is a very promising drug release strategy. Nanocarriers that respond to pH changes in tumor tissues can release drugs by changing the conformation or stability of the carrier when the environmental pH value changes through ionizable groups or acid-sensitive chemical bonds [14]. Despite the fact that some interesting smart pH-responsive nano-therapeutics have been reported, complicated chemical synthesis processes limit their clinical translation [15].

Carboxymethyl chitosan (CMCS), a carboxymethylated derivative of chitosan [16], is a promising charge-reversible material owing to its low toxicity, low immunogenicity, and good biocompatibility [17], together with the fact that (i) CMCS can be perfectly biodegraded in vivo, (ii) the CMCS coating provides a hydrophilic shell which offers a long circulation property after administration, (iii) and the abundant active groups of CMCS, including amino groups and carboxyl groups, can be functionalized with multitudinous ligands to strengthen the enhanced permeability and retention (EPR) effect, e.g., polymers such as poly (ethylene glycol) (PEG). More interestingly, as an amphoteric polysaccharide, CMCS is pH-sensitive because it contains both acidity (-COOH) and alkalinity (-NH_2_) [18,19]. Hence, CMCS is electronegative in the normal physiological environment, while is positive in the acidic tumor environment.

Among various nanocarriers, mesoporous silica nanoparticles (MSN) have garnered a great deal of attention as promising candidates for next-generation nanomedicines [20]. Several attractive features, such as stable mesoporous structures, large surface areas, tuneable pore sizes and volumes, and well-defined surface properties, have made them ideal for hosting molecules of various sizes, shapes, and functionalities [21]. Li et al. prepared platelet-membrane-coated MSN (MSNP) which could bind to collagen IV exposed to a surgical wound and efficiently inhibit post-surgical liver cancer relapse [22]. Cordeiro et al. reported redox-responsive MSN functionalized with a targeting ligand triantennary N-acetylgalactosamine (GalNAc) cluster and revealed the biopharmaceutical potential of MSN as a targeted drug carrier for therapeutic applications in liver diseases [23]. Since the mesoporous structure of bare MSN is open, the loaded drug is prone to leakage. To overcome this inherent defect, functionalized MSN with multitudinous gatekeepers can realize that the pores are open or closed and provide an alternative for controlled release [24,25]. Existing studies have evaluated the feasibility of CMCS as “gatekeepers” for MSN to achieve charge reversal and pH-responsive release in vitro, but these studies did not modify CMCS with the active targeting factor or evaluate its therapeutic efficacy and safety in vivo [26,27].

**Scheme 1:**
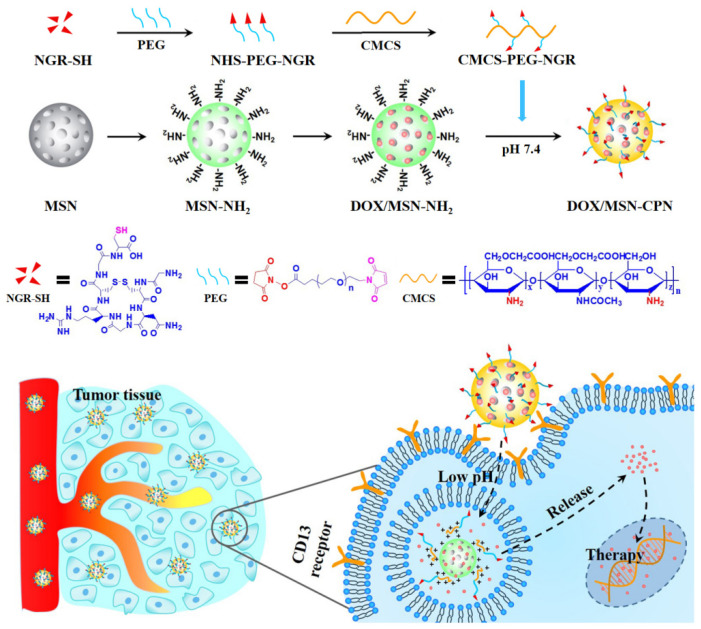
Schematic illustration of the procedure for DOX/MSN-CPN preparation. The modification of APTES endues MSN with a positive charge. The isoelectric point (pI) of CMCS-PEG-NGR is pH 6.81. In the blood circulation (pH 7.4), CMCS-PEG-NGR has a negative charge which can be coated on the surface of MSN-NH_2_ to prevent DOX release. While in the acidic tumor environment, especially the acidic environment of lysosomes in tumor cells, CMCS-PEG-NGR changes to a positive charge which can peel off the surface of MSN-NH_2_ to facilitate DOX release.

Hence, we prepared pegylated CMCS modified with an NGR peptide (CMCS-PEG-NGR, CPN) as a charge-reversible material to coat the MSN loaded with DOX (DOX/MSN-CPN) to achieve high accumulation and effective release of drugs at the tumor site (Scheme 1 and Figure S1). In this study, the therapeutic efficacy and safety of DOX/MSN-CPN for the treatment of hepatocellular carcinoma were evaluated both in vivo and in vitro. CPN kept an opposite charge to MSN in the preparation and blood circulation. CPN could be adsorbed on the surface of MSN which could coat the pores to prevent drug release, while CPN changed to the same charge as MSN when delivered to the tumor site. CPN was positively charged which could repel the MSN and it fell off to achieve drug-triggered release in the acidic environment of the tumor, especially the acidic environment of lysosomes in tumor cells. Furthermore, the CD13-targeted NGR peptide contained in CPN could increase the uptake of tumor cells through receptor-mediated endocytosis and thus improve the accumulation of drugs at tumor sites. Based on these characteristics, DOX/MSN-CPN not only had the ability of pegylated negative charge carriers to circulate in the body, but it could also promote cell uptake by positive charge and active targeting factors [28,29].

## 2. Materials and Methods

Tetraethoxysilane (TEOS), cetyltrimethylammonium bromide (CTAB), and 3-aminopropyltriethoxysilane (APTES) were obtained from Sinopharm Chemical Reagent Co., Ltd. (Shanghai, China). Mal-PEG_2000_-NHS was obtained from Bomei Biotechnology Co., Ltd. (Jiaxing, China). O-Carboxymethyl-chitosan (CMCS) (degree of deacetylation = 85%; degree of carboxymethyl substitution = 60%; average Mw = 50,000) was obtained from Haidebei Biotechnology Co., Ltd. (Jinan, China). DOX and 3-(4,5-dimethylthiazol-2-yl)-2,5-diphenyltetrazolium bromide (MTT) were purchased from Meilun Biology Technology Co., Ltd. (Dalian, China). cNGR with a cysteine on the C-terminal (sequence: GCNGRCGC) was synthesized by Apeptide Co. Ltd. (Shanghai, China). All of the other materials used in this study were of analytical reagent grade.

Female Kunming mice (6–8 weeks) were obtained from SPF Biotechnology Co., Ltd. (Beijing, China). All of the relevant animal experiments were performed under a protocol approved by the animal management rules of the Ministry of Health of the People’s Republic of China and the Animal Experiment Ethics Review Board of Shandong University.

### 2.1. Synthesis of CPN Conjugates

The charge-reversible pH-responsive material CPN was synthesized according to our previous work [30]. Briefly, cNGR-SH (15 mg, 0.02 mMol) and Mal-PEG_2000_-NHS (44 mg, 0.022 mMol) were dispersed in phosphate buffered saline (PBS, pH 7.4, 5 mL, 100 mM) and stirred for 1 h with the protection of nitrogen in an ice bath. Then, 50 mg CMCS (dissolved in 5 mL of PBS, pH 7.4) and 5 mg 1-(3-dimethylaminopropyl)-3-ethylcarbodiimide hydrochloride (EDC, dissolved in 1 mL of PBS, pH 7.4) were added into the above reaction solution under stirring. After 24 h, the reaction solution was dialyzed and lyophilized. Finally, the final product CPN was obtained. The structure of the CPN was verified by ^1^H NMR.

### 2.2. Acid-Base Titration of CPN

The NaCl (control) and polymer CPN were dissolved in 35 mL of deionized water at a final concentration of 30 mM, followed by a pH adjustment of 12 with 1 M NaOH. The diluted solution was titrated by the drop-by-drop addition of 1 M HCl solution, and a titration curve was obtained [31]. The experiments were conducted in triplicate.

### 2.3. Preparation of MSN-NH_2_ and FITC-MSN (MCM-41 Type)

The synthesis of MCM-41 was based on well-established published procedures [32]. Briefly, 0.5 g CTAB was added into 240 mL of water under ultrasonic conditions. To the solution, 1.75 mL of NaOH solution (2.00 M) was then added and stirred at 80 °C for 20 min. An amount of 2.5 mL of TEOS was added dropwise to the solution under vigorous stirring for 2 h at 80 °C. The produced particles were recovered by centrifugation (12,000 rpm, 20 min) and dried under vacuum for 24 h. To remove the surfactant CTAB residues, the dried particles were re-dispersed in 100 mL of ethanol and 20 mL of HCl (37% *v/v*) mixed solution and refluxed for 12 h. The reflux procedure was repeated twice to completely remove the template CTAB [27,33]. Finally, the solution was cooled to room temperature, centrifuged (12,000 rpm, 20 min), and washed several times with distilled water and ethanol to obtain the MCM-41-type MSN.

For modification of the MCM-41-type MSN by APTES, 100 mg MSN was dispersed in 50 mL of distilled water at 80 °C. Different amounts of APTES (20 μL, 40 μL, and 60 μL, respectively) were added dropwise to the solution under vigorous stirring for 2 h. Then, the reaction solution was centrifuged (12,000 rpm, 20 min) and dried under vacuum for 24 h to obtain amine-functionalized MSN (MSN-NH_2_).

The FITC-MSN was prepared according to well-established reported methods. FITC (3 mg) was dissolved in absolute ethanol (0.75 mL), APTES (60 μL) was added to the solution, and the mixture solution was kept in the dark overnight. Then, the following procedures were the same as the aforementioned method. Finally, FITC-MSN was obtained [34].

### 2.4. Preparation of DOX/MSN-CPN

Firstly, DOX-loaded MSN-NH_2_ (DOX/MSN-NH_2_) was prepared. Briefly, 10 mg of DOX was dissolved in 10 mL distilled water in dark. Then, 40 mg of MSN-NH_2_ powder was added to the above DOX solution in the dark at room temperature. After stirring for 24 h, the mixture was centrifuged (12,000 rpm, 20 min) and the supernatant was removed. The precipitate was washed with distilled water in order to remove the unloaded DOX and dried under vacuum for 24 h. The obtained red powder (DOX/MSN-NH_2_) was stored in the dark.

DOX/MSN-CPN was prepared by coating CPN to the surface of DOX/MSN-NH_2_ using electrical interaction. Briefly, 30 mg of DOX/MSN-NH_2_ (positive charge) was dispersed in 5 mL of PBS (pH 7.4). Then, CPN (dissolved in PBS, 7.4, negative charge) was dropped into the DOX/MSN-NH_2_ solution under stirring for 1 h. After stirring, the mixture was centrifuged (12,000 rpm, 20 min) and the supernatant was removed. The precipitate was washed with distilled water and dried under vacuum for 24 h. Finally, DOX/MSN-CPN was obtained. To find the optimum formulation, different weight ratios of DOX/MSN-NH_2_ and CPN (1:0, 90:1, 70:1, 50:1, 30:1, 10:1, 4:1, 2:1, and 1:1) were evaluated. DOX/MSN-CMCS or DOX/MSN-CMCS-PEG were also prepared by coating CMCS or CMCS-PEG to the surface of DOX/MSN-NH_2_ using the optimum weight ratio described above.

### 2.5. Physicochemical Characterization

Transmission electron microscopy (HT7700, Hitachi, Tokyo, Japan) was selected to visualize the morphology of DOX/MSN-NH_2_ and DOX/MSN-CPN. The DOX/MSN-NH2 or the DOX/MSN-CPN were dropped onto a copper grid and stained by 1% phosphotungstic acid. After air-drying, the samples were examined.

To test the size distribution, the average size was calculated. The zeta potential of the nanoparticles was analyzed by photon correlation spectroscopy (PCS) with a Delsa Nano C Particle Analyzer (Beckman Coulter, Bria, CA, USA). All of the measurements were made in triplicate in parallel. The average particle size was expressed in volume mean diameter and the reported value was expressed as mean ± standard deviation (SD) (*n* = 3).

To further verify the effective loading of DOX, ultraviolet—visible (UV-Vis) spectrophotometric analyses were performed. The absorbance of DOX was measured with a UV–Vis spectrophotometer at 480 nm. The drug loading efficiency (DL) and encapsulation efficiency (EE) were calculated from the following equations and the results were expressed as mean ± SD (*n* = 3).

DL (%) = (*W*_total drug input_ − *W*_total drug in supernatant_)/*W*_total nanoparticles_ × 100% EE (%) = (*W*_total drug input_ − *W*_total drug in supernatant_)/*W*_total drug input_ × 100%

N_2_ sorption was carried out with the help of a surface area and porosity analyzer (ASAP 2020, Micromeritics, Norcross, GA, USA). The Brunauer–Emmett–Teller (BET) method was applied to obtain the surface area. The Barrett–Joyner–Halenda (BJH) method was applied to determine the pore volume and pore size distribution. Pore diameter analysis was carried out by using the maximum of the pore size distribution curve.

### 2.6. In Vitro pH-Sensitivity

The pH-sensitivity of DOX/MSN-CPN was evaluated by measuring the zeta potential changes of DOX/MSN-CPN under different pH environments. Specifically, hydrochloric acid solution (1 M) was added drop-by-drop into a PBS buffer (pH 7.4) under stirring conditions, and appropriate amounts of PBS buffer were taken out at pH 7.1, 6.8, 6.4, 6.0, and 5.5 in turn for the experiment. DOX/MSN-CPN was dispersed in a PBS buffer with different pH values to achieve a final concentration of DOX of 100 μg/mL. At each pH value, the zeta potential was measured after incubation for 1 h.

### 2.7. In Vitro Biocompatibility

The biocompatibility of MSN-CPN was detected by MTT assay in vitro. Hepatocellular carcinoma cell strain HepG2 and macrophage strain RAW264.7 with a well growth state in the logarithmic growth stage were selected. The cells were seeded in 96-well plates at a density of 1×10^5^ cells/well and cultured overnight. After cell adherence, the corresponding samples were added to each well, and the final concentrations of MSN-CPN were 800, 400, 200, 100, 50, 25, 12.5, 6.25, 3.13, 1.56, and 0.78 μg/mL, with each concentration being repeated three times. The control group and the blank group were set. Then, the cells were placed in the incubator and cultured for 48 h, followed by adding 20 μL of MTT solution (5 mg/mL) into each well and they continued to incubate for 4 h. After incubation, the supernatant was discarded and 150 μL of DMSO was added to each well. Finally, the absorbance was measured at 570 nm and the cell viability was calculated.

### 2.8. In Vitro Drug Release

Regarding the in vitro release of DOX from the DOX solution (dissolved in PBS 7.4), DOX/MSN-NH_2_ and DOX/MSN-CPN were evaluated using the dialysis bag diffusion technique. Typically, 1 mL of DOX solution (1 mg/mL), DOX/MSN-NH_2_ (DOX concentration: 1 mg/mL), and DOX/MSN-CPN (DOX concentration: 1 mg/mL) were placed in a pre-swelled dialysis bag with a 3.5 kDa molecular weight cutoff. The bags were placed into a tube with 10 mL of PBS solution (pH 5.0, pH 6.0, or pH 7.4) at 37 ± 0.5 °C with a horizontal shaking speed of 100 rpm. At predetermined time points, the sample was collected and replaced with 10 mL of fresh medium. The concentration of released DOX was determined by the UV–Vis spectrophotometry method. The experiments were performed in triplicate. The release rate was calculated from the following equation and the results were expressed as mean ± SD (*n* = 3).

$$\mathrm{Release}\;\mathrm{rate}\;(\%)=Q_{n}/w\;\times \;100\%=\sum_{i=1}^{n}C_{i}V/w\;\times \;100\%$$

where Q_n_ is the accumulated DOX release mass; w is the total DOX content of the release sample; *C_i_* is the DOX concentration in the release medium at each predetermined time point; and *V* is the volume of the release medium.

### 2.9. Cell Culture

Human hepatocellular carcinoma cells strain HepG2 and mouse hepatocarcinoma cells strain H22, overexpression of CD13 [13], were incubated in an RPMI 1640 medium supplemented with 10% fetal bovine serum (FBS), streptomycin, and penicillin (1%). Human normal hepatocytes strain HL-7702 was incubated in an RPMI 1640 medium supplemented with 10% fetal bovine serum (FBS), streptomycin, and penicillin (1%). Macrophage strain RAW264.7 was incubated in a DMEM medium supplemented with 10% fetal bovine serum (FBS). All of the cells were cultured in a 37 °C incubator with 5% CO_2_.

### 2.10. In Vitro Cell Uptake of DOX/MSN-CPN

The in vitro cell uptake of DOX/MSN-CPN was captured by a fluorescence microscope (BX40, Olympus, Tokyo, Japan) and quantified by flow cytometry (Accuri C6 Plus, BD Biosciences, Franklin Lakes, NJ, USA). The HepG2 cells were seeded into 12-well plates and cultured in a 37 °C incubator with 5% CO_2_ overnight. The cells were treated with a fresh RPMI 1640 medium supplemented with 10% FBS, 1% streptomycin, and penicillin containing DOX/MSN-CPN (DOX concentration was 20 μg/mL) for 1 h and 4 h. Then, the cells were washed twice with cold PBS and the fixed cells with 4% paraformaldehyde were examined under a fluorescence microscope. DOX solution, DOX/MSN-CMCS-PEG, and DOX/MSN-CPN+NGR (5 × 10^5^ cells were preincubated with 1 mg/mL free NGR for 1 h) were set as control groups. To quantify the cellular uptake of DOX solution, DOX/MSN-CMCS-PEG, DOX/MSN-CPN, and DOX/MSN-CPN+NGR, the cells were treated as mentioned above and evaluated by flow cytometry.

### 2.11. In Vitro Cell Uptake of FITC-MSN-CPN

The in vitro cell uptake of FITC-MSN-CPN was quantified by flow cytometry (CytoFLEX S, Beckman Coulter Life Sciences, Bria, CA, USA). Human hepatocellular carcinoma cell strain HepG2 and human normal hepatocytes strain HL-7702 were seeded into 12-well plates and cultured in a 37 °C incubator with 5% CO_2_ overnight. The cells were treated with fresh RPMI 1640 medium supplemented with 10% FBS, 1% streptomycin, and penicillin containing FITC-MSN-CPN (FITC concentration was 50 μg/mL) for 1 h and 4 h. Then, the cells were washed twice with cold PBS and collected for evaluating by flow cytometer. FITC-MSN-CMCS-PEG and FITC-MSN-CPN+NGR (free NGR was preincubated for 1 h) were set as control groups.

### 2.12. Ex Vivo Animal Imaging

The animal imaging experiment was carried out on H22 tumor-bearing mice using an in vivo real-time fluorescence imaging system (IVIS) spectrum (Cailper PerkinElemer, Waltham, MA, USA). A total of 1 × 10^6^ H22 cells were injected into the right axillary space of the mice to establish the H22-tumor-bearing mice model. The mice were given intravenous (*i.v.*) injections of DOX or DOX/MSN-CPN at the DOX dose of 10 mg/kg when the tumors grew to approximately 500 mm^3^. After 2 h, 8 h, and 24 h of injection, three mice in each group were sacrificed and the tumors and main organs (the heart, liver, spleen, lungs, and kidneys) were separated from the mice and put on a black paperboard. Fluorescence of the tumors and organs was observed by IVIS at appropriate wavelengths (excitation wavelength of 465 nm and emission wavelength of 583 nm). The fluorescence intensity was quantified to evaluate the drug distribution in tumors and different organs.

### 2.13. In Vivo Tumor Inhibition

An in vivo tumor growth inhibition study was employed to evaluate the antitumor effects of DOX/MSN-CPN. Briefly, H22 cells (1 × 10^6^) were subcutaneously injected into the right axilla of the mice. The mice were randomly separated into five groups (six mice per group) when the tumors grew to approximately 100 mm^3^. Different groups of mice were treated with (1) normal saline (NS) injection (*i.v.*); (2) DOX solution (10 mg/kg; *i.v.*); (3) MSN-CPN (the same dosage as DOX/MSN-CMCS without DOX, *i.v.*); (4) DOX/MSN-CMCS (10 mg/kg; *i.v.*); and (5) DOX/MSN-CPN (10 mg/kg; *i.v.*) at 1, 4, 7, 10, and 14 days, respectively. Tumor volumes and body weights were measured every 3 days. On day 22, the mice were sacrificed, and the tumors were photographed.

### 2.14. Histological Analysis

MSN-CMCS (the same dosage as in tumor inhibition studies) was injected into five female Kunming mice (6–8 weeks) via the tail vein at 1, 4, 7, 10 and 14 days. NS was used as a control reagent.

### 2.15. Statistical Analysis

All of the data are presented as means ± SD. Comparisons between the groups were analyzed by a one-tailed Student’s *t*-test. The *p* value was calculated with the help of GraphPad Prism 8. Statistically significant differences were considered when the *p* values were less than 0.05.

## 3. Results and Discussion

### 3.1. Synthesis of CPN

**Figure 1:**
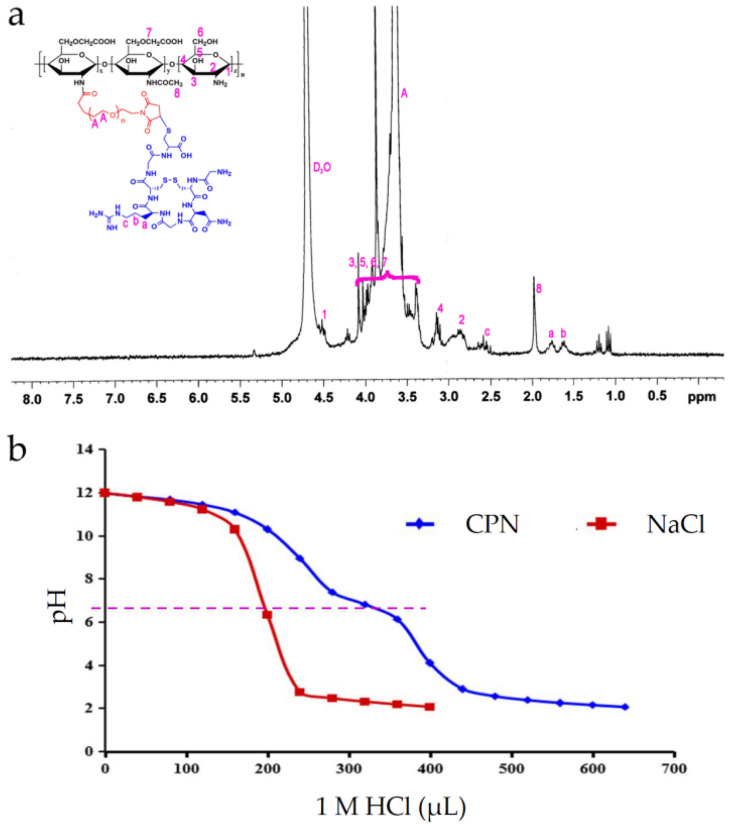
(**a**) ^1^H NMR of CPN in D_2_O. The characteristic proton peaks of CMCS (black), PEG (red), and NGR (blue) confirmed the successful synthesis of CPN and (**b**) the titration profile of NaCl and CMCS-PEG-NGR. The pI of CMCS-PEG-NGR is pH 6.81.

CMCS contained many active groups, such as amino groups and carboxyl groups, which can be easily modified with other functional ligands. Many studies have used CMCS as the backbone material to synthesize multifunctional drug conjugates for tumor-targeted drug delivery and controlled release [30,35]. In this study, the amino groups of CMCS were employed to link with PEG-NGR by AN amide reaction to endow CMCS with a better hydrophilic property and an active targeting function. The ^1^H NMR of CPN in D_2_O is shown in Figure 1a. The peak at 3.616 ppm (marked A) came from the PEG. The peaks at 1.607, 1.760, and 2.549 ppm (marked as a, b, and c, respectively) came from the NGR. These peaks indicated the successful synthesis of CPN. The modification rate of NGR was 13.91% which was calculated by the ratio of the peak area of the characteristic peak at 1.973 ppm (18,947.91, -OCH3, CMCS) to the peak area of the characteristic peak at 1.607 ppm (11,715.63, -CH2-, NGR).

### 3.2. Acid–Base Titration of CPN

After the successful synthesis of CPN, the pI of CPN was tested by acid–base titration. Since the backbone material CMCS can change its charge when the environmental pH changes across its pI, it is necessary to test whether the pI of CPN was located between the physiological pH and the acidic tumor environment. To evaluate the pI of CPN, the acid–base titration method was employed, and the profile is presented in Figure 1b. Because CPN contained ionizable alkaline groups and protonizable acidic groups, it had a great buffering capacity with a buffering range between pH 5.0 and 7.0 and it is shown in Figure 1b, while the NaCl solution had no buffering ability. Calculated from the stoichiometric point of the titration jump, the pI is 6.81, which was located between the physiological pH (pH 7.4) and the acidic tumor environment (pH 6.0), especially the acidic environment of lysosomes in tumor cells (pH 5.0). This result indicated that when the environmental pH was above 6.81, CPN showed as being negatively charged which can be absorbed on the surface of positively charged nanoparticles. When the environmental pH was below 6.81, CPN showed as being positively charged, which could peel off gradually because of the electrical interaction.

### 3.3. Characterization of the DOX/MSN-CPN

To absorb the negatively charged CMCS to the surface of MSN at a physiological pH, the initial MSN should be positively charged. Therefore, after the successful preparation of MCM-41-type MSN, APTES which contained many amino groups was linked to the surface of MSN to obtain MSN-NH_2_. The effect of the amount of APTES on MSN-NH_2_ is shown in Table 1. APTES did not show a significant effect on the size of MSN-NH_2_, while the zeta potential of MSN-NH_2_ increased with the increase in the amount of APTES. It has been demonstrated that nanocarriers are more stable when the zeta potential is over 30 mV [36]. Thus, 60 μL of APTES was selected as the optimum amount and was used in the following studies. The obtained MSN-NH_2_ was further verified through low-angle X-ray diffraction measurements (Figure S2). MSN-NH_2_ exhibited a very intense diffraction peak at 2θ values of 2.1° for the 100 reflection plane which indicated the presence of mesoporosity in MSN-NH_2_.

**Figure 2:**
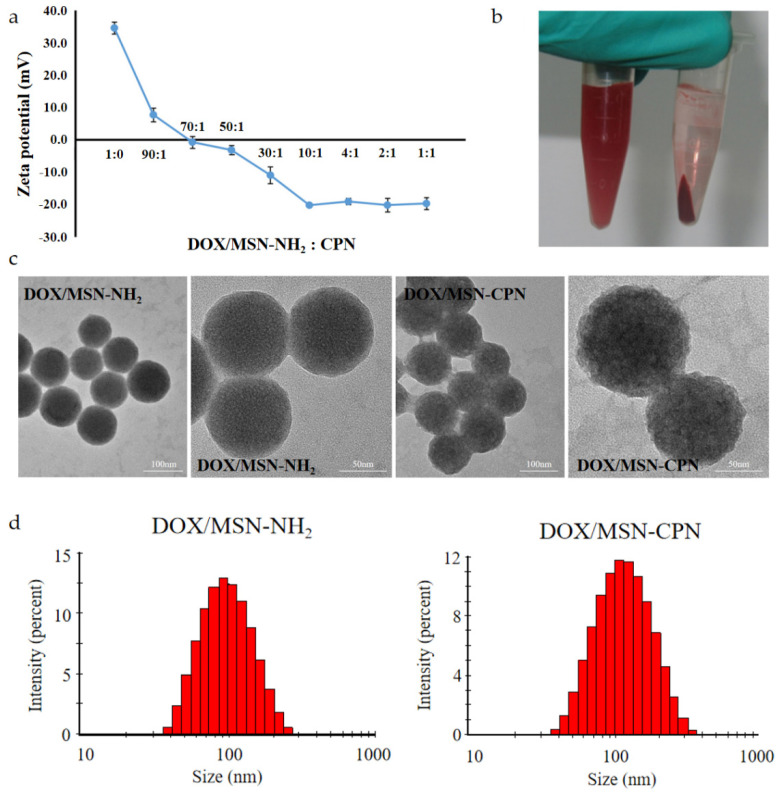
(**a**) The impact of weight ratio of DOX/MSN-NH_2_ and CPN on zeta potential; (**b**) the appearance of DOX/MSN-CPN before and after centrifugation; (**c**) the morphology of DOX/MSN-NH_2_ and DOX/MSN-CPN in transmission electron microscopy; and (**d**) the size distribution of DOX/MSN-NH_2_ and DOX/MSN-CPN.

Another important factor in DOX/MSN-CPN preparation is the ratio of DOX/MSN-NH_2_ and CPN. As shown in Figure 2a, the zeta potential changed from positively charged to negatively charged with the increase in CPN, which indicated that CPN was absorbed to the surface of DOX/MSN-NH_2_ and covered the positive charge. When the ratio of DOX/MSN-NH_2_ and CPN was 10:1, adding more CPN did not change the zeta potential of DOX/MSN-CPN. Therefore, this ratio was selected as the optimum in the following experiments.

The final product DOX/MSN-CPN showed a dark red color which is shown in Figure 2b and Figure S3. After centrifugation, the supernatant was colorless and transparent, while the precipitate had a dark red color. These phenomena indicated that DOX was completely loaded in the mesoporous structure of DOX/MSN-CPN. The characteristics of DOX/MSN-NH_2_ and DOX/MSN-CPN (*n =* 3) are showed in Table 2. Because of the CPN coating, the particle size increased from 104.24 ± 4.32 nm to 125.01 ± 1.52 nm (Figure 2d). The zeta potential changed from 35.23 ± 1.18 mV to −20.2 ± 0.45 mV. The TEM images of DOX/MSN-NH_2_ and DOX/MSN-CPN are showed in Figure 2c. Both the DOX/MSN-NH_2_ and DOX/MSN-CPN had spherical shapes with a uniform size distribution. Compared with DOX/MSN-NH_2_, the surface of DOX/MSN-CPN was relatively rough, which indicated that CPN was successfully wrapped on the surface of DOX/MSN-NH_2_.

**Figure 3:**
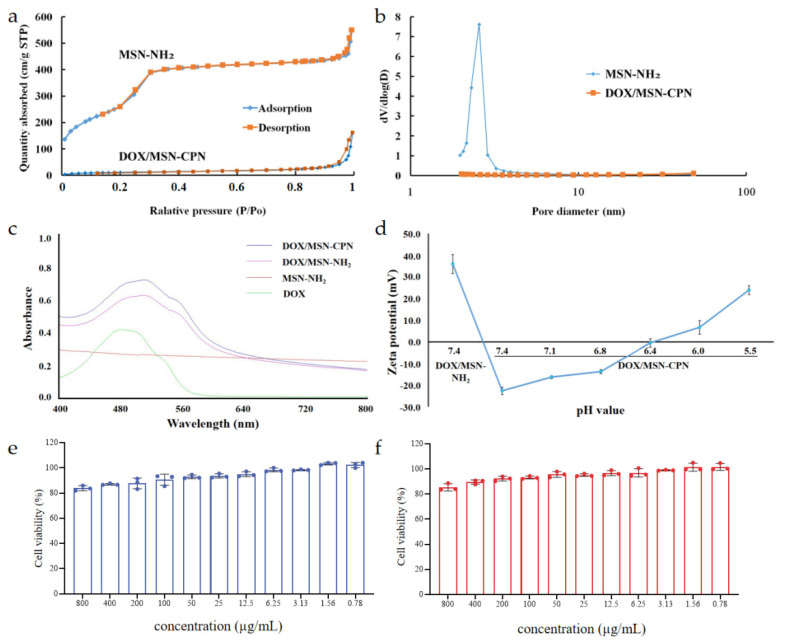
(**a**) Nitrogen physisorption measurements of MSN-NH_2_ and DOX/MSN-CPN; (**b**) the pore diameter of MSN-NH_2_ and DOX/MSN-CPN; (**c**) the UV–Vis spectrophotometry of DOX, MSN-NH_2_, DOX/MSN-NH_2_, and DOX/MSN-CPN; (**d**) the zeta potential changes of DOX/MSN-CPN with the decrease in pH from 7.4 to 5.5; (**e**) the cell viability of MSN-CPN and HepG2 cells co-incubated for 48 h; (**f**) and the cell viability of MSN-CPN and RAW264.7 cells co-incubated for 48 h.

The porosity of MSN-NH_2_ and DOX/MSN-CPN was characterized by N_2_ sorption, and the results are shown in Figure 3a. According to the classification of the International Union of Pure and Applied Chemistry (IUPAC), both of the N_2_ adsorption–desorption isotherms were typical type IV isotherms, indicating the typical characteristics of mesoporous silica. The pore size distribution (inset) of MSN-NH_2_ was 2.87 nm obtained by a pore size distribution curve (Figure 3b). In contrast, the mesoporous structure of DOX/MSN-CPN disappeared which indicated that the drug loading or the coating of CPN blocked the pores of MSN-NH_2_. The summary of nitrogen sorption measurements is shown in Table 3. The BET surface area and BJH adsorption cumulative volume of the pores of DOX/MSN-CPN were significantly decreased (*p* < 0.05) compared to MSN-NH_2_ which demonstrated that DOX was loaded inside the cores and CPN could absorb and block the cores as a “gatekeeper”.

To further confirm the successful loading of DOX, the visible absorption spectrums of DOX, MSN-NH_2_, DOX/MSN-NH_2_, and DOX/MSN-CPN were scanned, and the results are shown in Figure 3c. MSN-NH_2_ did not exhibited an absorption peak between the wavelengths of 400 and 800 nm. DOX, DOX/MSN-NH_2_, and DOX/MSN-CPN showed similar absorption curves, indicating successful DOX loading. Compared with DOX, the maximum absorption wavelength of DOX/MSN-NH_2_ and DOX/MSN-CPN transferred from 480 nm to 509 nm, which may be caused by the effect of MSN carriers.

### 3.4. In Vitro pH-Sensitivity

The pH-sensitivity was the key factor of DOX/MSN-CPN. In this study, the zeta potential change at different pH values was evaluated. As shown in Figure 3d, the zeta potential of DOX/MSN-CPN increased with the decrease in environmental pH from 7.4 to 5.5. When the environmental pH was below 6.4, the zeta potential changed to positive. A possible reason was that CPN showed positive charge when the pH was below its pI. Because the inner core (MSN-NH_2_) was also positively charged, CPN could peel off gradually due to electrical repulsion and the original positive surface was exposed. These results show that when DOX/MSN-CPN accumulated in the acidic tumor microenvironment or acidic lysosomes through the blood circulation, the gatekeeper CPN could responsively “open” the pores and facilitate drug release.

### 3.5. In Vitro Biocompatibility

In order to verify the feasibility of MSN-CPN as a nanocarrier, the biosafety of MSN-CPN was studied in vitro. The cytotoxicity of MSN-CPN was detected by MTT assay in vitro. Hepatocellular carcinoma cell strain HepG2 and macrophage strain RAW264.7 were selected for cell experiments. For both types of cells, MSN-CPN showed no significant biotoxicity at concentrations ranging from 0.78 µg/mL to 800 µg/mL. After 48 h of incubation, even at a high concentration of 800 µg/mL, the survival rate of HepG2 was still up to 83.88 ± 2.01% and the survival rate of RAW264.7 was still up to 85.38 ± 2.88%. These results indicate that MSN-CPN has excellent biocompatibility.

### 3.6. In Vitro Drug Release

**Figure 4:**
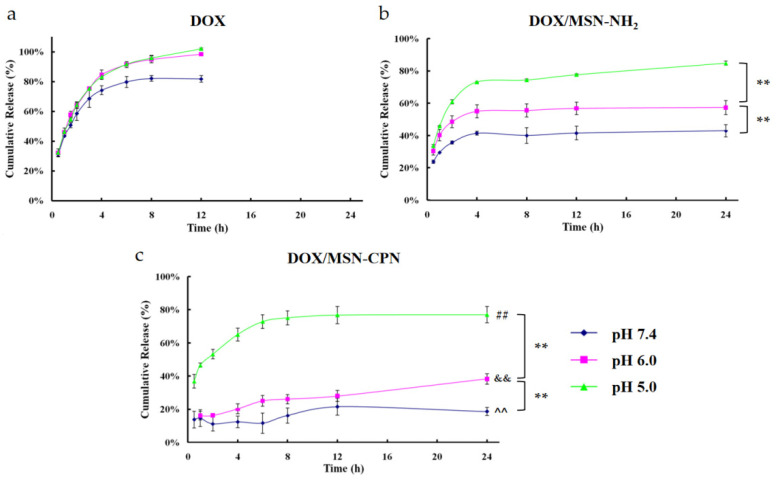
The in vitro release profile of the DOX solution (**a**); DOX/MSN-NH_2_ (**b**); and DOX/MSN-CPN (**c**) in 24 h. The release media is PBS (pH = 7.4, 6.0 or 5.0). [^^^^ *p <* 0.01, compared to DOX/MSN-NH_2_ (pH 7.4); ^&&^ *p* < 0.01, compared to DOX/MSN-NH_2_ (pH 6.0); ^##^ *p* < 0.01, compared to DOX/MSN-NH_2_ (pH 5.0); ** *p* < 0.01, compared between the two groups were shown in parentheses].

To evaluate the pH-sensitive drug release of DOX/MSN-CPN, the in vitro release profiles of DOX solution, DOX/MSN-NH_2_, and DOX/MSN-CPN were investigated in PBS with different pH values. The in vitro release behavior was shown as a cumulative release percentage in Figure 4. The release of DOX from the DOX solution was fast at all pH values. In contrast, the release of DOX from DOX/MSN-NH_2_ and DOX/MSN-CPN showed an obvious pH-sensitive property. In both DOX/MSN-NH_2_ and DOX/MSN-CPN groups, DOX released much slower at pH 7.4 than at pH 6.0 (*p* < 0.01). Moreover, DOX released much slower at pH 6.0 than at pH 5.0 (*p* < 0.01). For the DOX/MSN-NH_2_ group, the pH-sensitive release profile was mainly caused by the solubility difference of DOX at pH 7.4, 6.0, or 5.0. The solubility and dissolution rate of DOX increased with the decrease in the pH value. Thus, a faster diffusion rate occurred at a lower pH level when DOX was diffused out of the DOX/MSN-NH_2_ cores, which resulted in the pH-sensitive release of DOX/MSN-NH_2_. For the DOX/MSN-CPN group, CPN served as a pH-sensitive “gatekeeper” to control DOX release. At pH 7.4, the cores were completely blocked by CPN and DOX release was inhibited. Thus, the release of DOX from DOX/MSN-CPN (pH 7.4) was significantly slower than that from DOX/MSN-CPN (pH 6.0, *p* < 0.01), DOX/MSN-CPN (pH 5.0, *p* < 0.01), and DOX/MSN-NH_2_ (pH 7.4, *p* < 0.01). At pH 6.0, DOX release was still prevented because CPN was only partially shed. Therefore, the release of DOX from DOX/ MSN-CPN (pH 6.0) was significantly slower than that from DOX/ MSN-CPN (pH 5.0, *p* < 0.01) and DOX/MSN-NH_2_ (pH 6.0, *p* < 0.01). At pH 5.0, CPN peeled off from the surface of THE nanoparticles and the cores opened. DOX showed a fast release profile that was similar to DOX/MSN-NH_2_. These results indicate that CPN was a lysosome pH-sensitive “gatekeeper” which can achieve tumor-environment-triggered drug release.

### 3.7. In Vitro Cell Uptake

**Figure 5:**
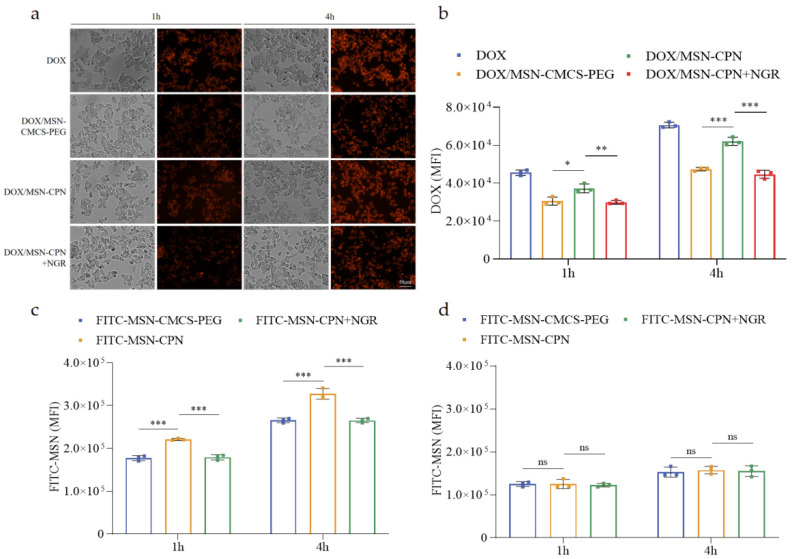
Fluorescence microscopy images (**a**) and flow cytometry test results (**b**) of DOX, DOX/MSN-CMCS-PEG, DOX/MSN-CPN or DOX/MSN-CPN+NGR and HepG2 cells co-incubated for 1 h and 4 h; flow cytometry test results of FITC-MSN-CMCS-PEG, FITC-MSN-CPN or FITC-MSN-CPN+NGR and HepG2 cells (**c**) or HL-7702 cells (**d**) co-incubated for 1 h and 4 h. (* *p* < 0.1; ** *p* < 0.01; *** *p* < 0.001; ns, no significance).

Given that DOX has its own fluorescence, we first investigated the targeting delivery ability of DOX/MSN-CPN to the packaged drug. CD13-positive cell strain HepG2 was used for the cellular uptake of DOX/MSN-CPN. As shown in Figure 5a,b, the internalizations of DOX, DOX/MSN-CMCS-PEG, and DOX/MSN-CPN were both time dependent because the fluorescence intensity measured by fluorescence microscopy images and flow cytometry changed with time. At 1 h and 4 h, the fluorescence intensity of the DOX/MSN-CPN group was higher than that of the DOX/MSN-CMCS-PEG group. For competitive inhibition assays, HepG2 cells were incubated with free NGR in advance to saturate CD13 and then with DOX/MSN-CPN (DOX/MSN-CPN+NGR). The fluorescence intensity of the DOX/MSN-CPN group was higher than that of the DOX/MSN-CPN+NGR group, because most of the CD13 on the HepG2 cells had already bound to free NGR and could not interact with NGR on DOX/ MSN-CPN.

To further rule out the potential leakage of fluorescent molecules and other cellular internalization mechanisms distinct from receptor-mediated endocytosis, MSN covalently combined with FITC (FITC-MSN) was prepared. CD13-positive cell strain HepG2 (Figure 5c) and CD13-negative cells strain HL-7702 (Figure 5d) were used for the cellular uptake of FITC-MSN-CPN. The same as the cellular uptake of DOX/MSN-CPN, the fluorescence intensity of the FITC-MSN-CPN group was significantly better than that of the FITC-MSN-CMCS-PEG group (*p* < 0.001) and the FITC-MSN-CPN+NGR group (*p* < 0.001) at 1 h and 4 h. However, internalization of all of the groups in HL-7702 cells was less than that in the HepG2 cells and there were no significant differences among the experimental groups. These results suggest that NGR modification could increase cell uptake through CD13-mediated internalization.

### 3.8. Ex Vivo Animal Imaging

**Figure 6:**
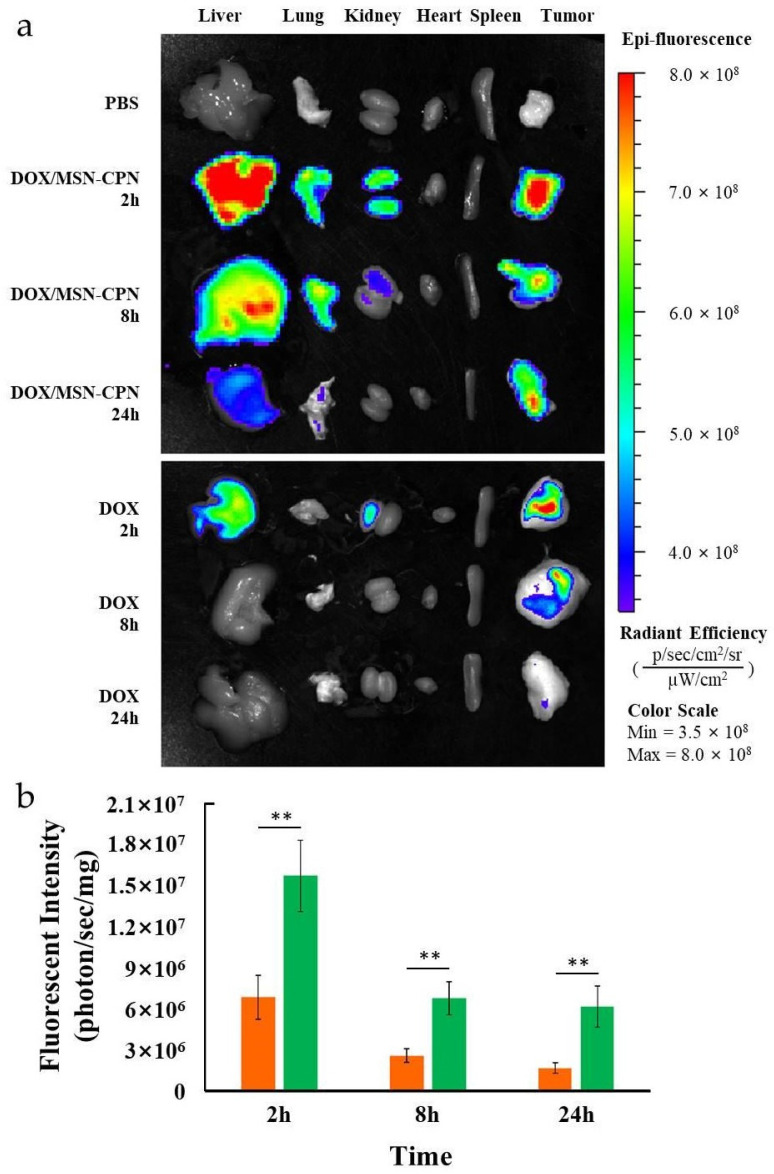
In vivo distribution of DOX in H22-tumor-bearing mice. (**a**) Ex vivo imaging of the main tissues and the tumor after injection with different formulations for 2, 8, and 24 h and (**b**) the ex vivo imaging fluorescence signal intensity of DOX solution (Orange) and DOX/MSN-CPN (Green) in the tumor at 2, 8, and 24 h. (*n =* 3, ** *p* < 0.01).

To characterize the DOX distribution in different tissues, DOX/MSN-CPN was administered to H22-tumor-bearing mice via intravenous injection and the fluorescence signal was acquired by an IVIS spectrum imaging system. A DOX solution at the same dosage was set as the control group. Unexpectedly, the fluorescence signal of DOX could not be detected in the living mice body. Therefore, the ex vivo imaging of different tissues was employed to evaluate the DOX distribution. As shown in Figure 6, the DOX group could rapidly accumulate in the liver and tumor site at 2 h after injection, but the fluorescence intensity decreased rapidly afterwards. Twenty-four h after injection, the imaging signal could not be detected. In contrast, the DOX/MSN-CPN group displayed a significantly higher signal intensity in the liver, lungs, kidneys, and tumor (*p* < 0.01). Even 24 h after injection, the imaging signal of the tumor tissue maintained a high signal brightness. In order to better compare the tumor signal difference between the DOX group and the DOX/MSN-CPN group, the signal intensity was quantified and the results are shown in Figure 6b. At each time point, the signal intensity of the DOX/MSN-CPN group was significantly higher than that of the DOX group, which indicated that DOX/MSN-CPN could efficiently deliver DOX to the tumor tissue and DOX could accumulate in the tumor tissue for a long time. Based on these results, it could be speculated that DOX/MSN-CPN could improve the therapeutic efficiency of DOX after administration.

### 3.9. In Vivo Tumor Inhibition

**Figure 7:**
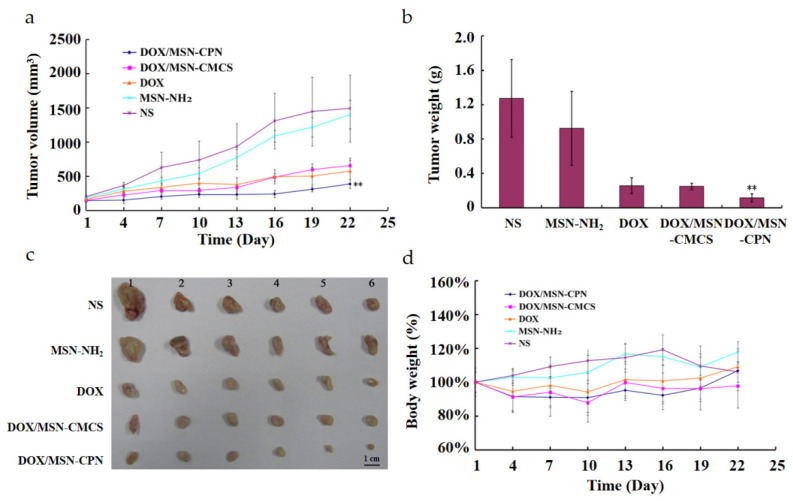
In vivo tumor growth inhibition study of NS, MSN-NH_2_, DOX solution, DOX/MSN-CMCS and DOX/MSN-CPN. (**a**) The tumor volume profile of different groups over 22 days; (**b**) the tumor weight of the different groups at the 22 days; (**c**) The tumor images of different groups at 22 days; (**d**) the body weight changes of the different groups over 22 days. (*n =* 6, ** *p* < 0.01 compared with DOX solution and DOX/MSN-CMCS).

The antitumor efficacy in vivo was evaluated in H22-tumor-bearing mice. All of the results are shown in Figure 7. Compared to the NS group, MSN-NH_2_ did not show favorable antitumor effects in vivo, as shown in Figure 7a. In contrast, the DOX, DOX/MSN-CMCS, and DOX/MSN-CPN groups all showed significant antitumor effects compared to the NS group (*p* < 0.01). Among these three groups, DOX/MSN-CPN showed the highest tumor inhibition (*p* < 0.01). To better observe the tumor inhibition, all of the tumors were separated and weighed at 22 day, Figure 7b,c. The tumor weight of the DOX/MSN-CPN group was significantly lighter than any of the other groups (*p* < 0.01) indicating that DOX/MSN-CPN had the best tumor therapeutic effect. This tumor inhibition could also be directly observed in Figure 7c. The reason may be that DOX/MSN-CPN could optimize the biodistribution of free drugs after injection and more DOX accumulated in the tumor area due to the passive targeting and active targeting of nanoparticles. Otherwise, the lysosome-triggered release properties of DOX/MSN-CPN could further increase tumorcelltargeted delivery, thus increasing the therapeutic effect. The safety of DOX/MSN-CPN was evaluated by weighing the body weight of the mice over 22 days. The DOX/MSN-CPN group did not show significant body weight loss over 22 days and the body weight of the DOX/MSN-CPN group did not show significant differences compared to the NS group. The possible reason was that DOX was released mainly after the absorption to lysosomes, which decreases the drug distribution of DOX in normal tissues. Therefore, the toxicity of DOX/MSN-CPN was decreased.

### 3.10. Histological Analysis

**Figure 8:**
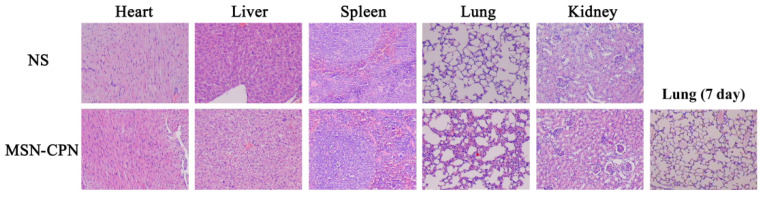
Representative organ histology for NS- (**top row**) and MSN-CPN (**bottom row**)-injected mice. The last image in the bottom row represents the lung histological image of the MSN-CPN group after treatment for 7 days.

To preliminarily evaluate the safety of MSN-CPN, histological analysis after in vivo antitumor inhibition studies was employed in this study. Histological images of major organs (including the heart, liver, spleen, lungs, and kidneys) were used to determine whether or not MSN-CPN caused tissue lesions. As shown in Figure 8, the MSN-CPN group (bottom row, Figure 8) showed no visual pathological changes compared to the control (top row, Figure 8) in the heart, liver, spleen, and kidneys. The histological image of the lung showed reversible inflammatory cell infiltration compared to the NS group, which might be caused by the repeated administration of MSN-CPN. Seven days after treatment, inflammatory cell infiltration basically disappeared. These results indicate that MSN-CPN has good biocompatibility.

## 4. Conclusions

The pathogenesis of liver cancer is not completely clear, and it is characterized by a short course of disease, rapid onset, and difficult diagnosis and treatment. Most patients with liver cancer cannot be diagnosed until they are in an advanced stage, which means surgical resection and orthotopic liver transplantation are no longer applicable [37]. For these patients with liver cancer in an advanced stage, systemic chemotherapy remains the only therapy. However, systemic chemotherapy’s efficacy is not expected due to its short half-life, non-specific distribution, and dose-limited toxicity [38]. Nano drug delivery systems provide a promising strategy for enhancing the efficacy of antineoplastic drugs.

In summary, a CD13-mediated pegylated CMCS-capped MSN was designed and prepared as a platform for drug-targeted delivery and triggered release. Compared with traditional lipid nanocarriers, DOX/MSN-CPN could significantly increase drug loading efficiency and greatly reduce the number of carriers used [39]. Compared with the chemical bonding of drugs to MSN, the preparation process of encapsulation using a charge-reversible material was simpler and more universal [40]. The multifunctional DOX/MSN-CPN could effectively accumulate in the cancer area and could be actively absorbed by the cancer cells. Moreover, DOX/MSN-CPN showed pH-sensitive release of DOX and effectively tumor growth inhibition in vivo. These results demonstrate that DOX/MSN-CPN could achieve the high accumulation and effective release of drugs at the tumor site, which may be a therapeutic strategy for future clinical applications in the systematic treatment of patients with advanced liver cancer since it compensates for the deficiencies associated with current systemic chemotherapy strategies.

## Figures and Tables

**Table 1:** The effect of different amounts of APTES on MSN-NH_2_ (*n =* 3).

	1	2	3
APTES (µL)	20	40	60
Zeta potential (mV)	−0.26 ± 0.52	26.40 ± 1.87	35.23 ± 1.18
Size (nm)	104.15 ± 12.07	99.45 ± 8.10	101.91 ± 10.83

**Table 2:** Characteristics of DOX/MSN-NH_2_ and DOX/MSN-CPN (*n =* 3).

	Size (nm)	PDI	Zeta Potential (mV)	EE (%)	DL (%)
DOX/MSN-NH_2_	104.24 ± 4.32	0.195 ± 0.019	35.23 ± 1.18	52.52 ± 0.87	11.61 ± 0.17
DOX/MSN-CPN	125.01 ± 1.52	0.213 ± 0.015	−20.20 ± 0.45	—	10.55 ± 0.15

**Table 3:** The surface area, volume of pores, and pore diameter of MSN and MSN-CPN.

	BET Surface Area (cm^2^/g)	BJH Adsorption Cumulative Volume of Pores (cm^3^/g)	BJH Desorption Average Pore Diameter (nm)
MSN-NH_2_	1185.12	0.89	2.87
DOX/MSN-CPN	41.07	0.25	—

## Data Availability

Raw data are available from the corresponding authors upon request.

## Supplementary Materials

The following supporting information can be downloaded at: https://www.mdpi.com/article/10.3390/pharmaceutics15020426/s1. Figure S1: Schematic illustration of the procedure for DOX/MSN-CPN preparation; Figure S2: Low-angle XRD patterns of MSN-NH_2_; Figure S3: The visual appearance of DOX/MSN-NH_2_ (left) and DOX/MSN-CPN (right).
