# Supplementary material for: CD13-Mediated Pegylated Carboxymethyl Chitosan-Capped Mesoporous Silica Nanoparticles for Enhancing the Therapeutic Efficacy of Hepatocellular Carcinoma
Source: Pharmaceutics. 2023 Jan 28;15(2):426. doi: 10.3390/pharmaceutics15020426 (PMC9962034; doi:10.3390/pharmaceutics15020426)
Supplement: Supplementary file 1 [file pharmaceutics-15-00426-s001.zip › pharmaceutics-1969057-supplementary.pdf]

Supplementary Information:

CD13-Mediated Pegylated Carboxymethyl Chitosan-Capped Mesoporous Silica Nanoparticles for Enhancing the Therapeutic Efficacy of Hepatocellular Carcinoma

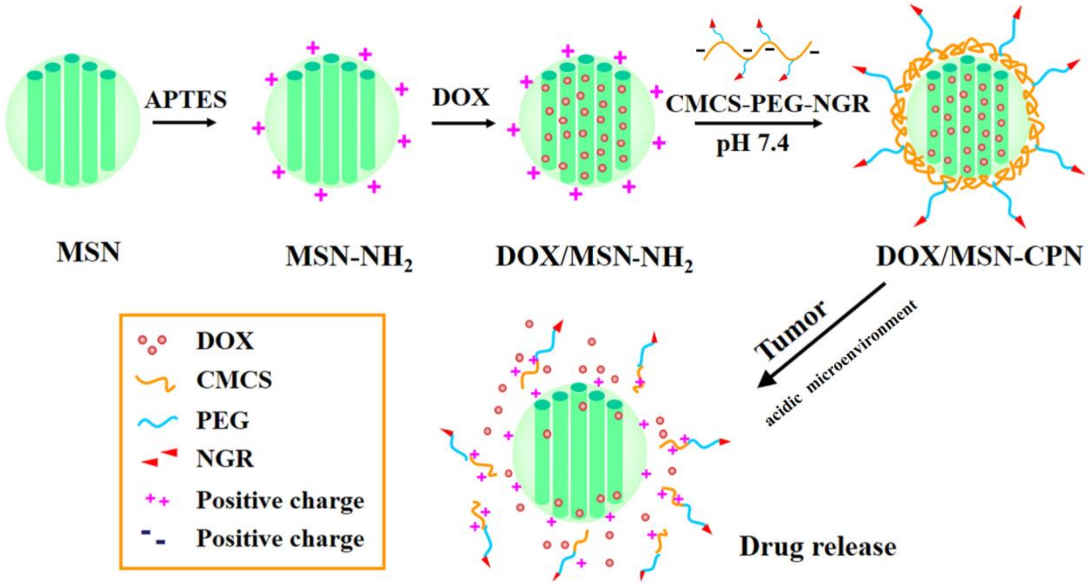

Figure S1 Schematic illustration of the procedure for DOX/MSN-CPN preparation.

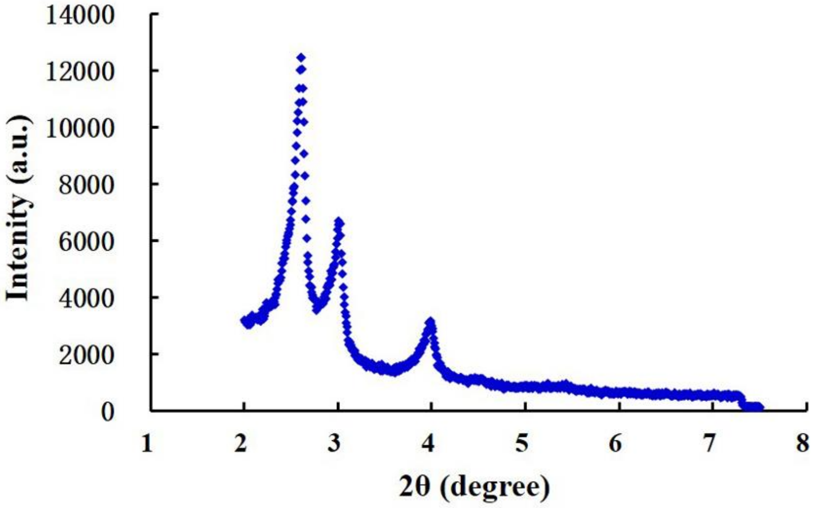

Figure S2 Low angle XRD paterns of MSN-NH$_2$.

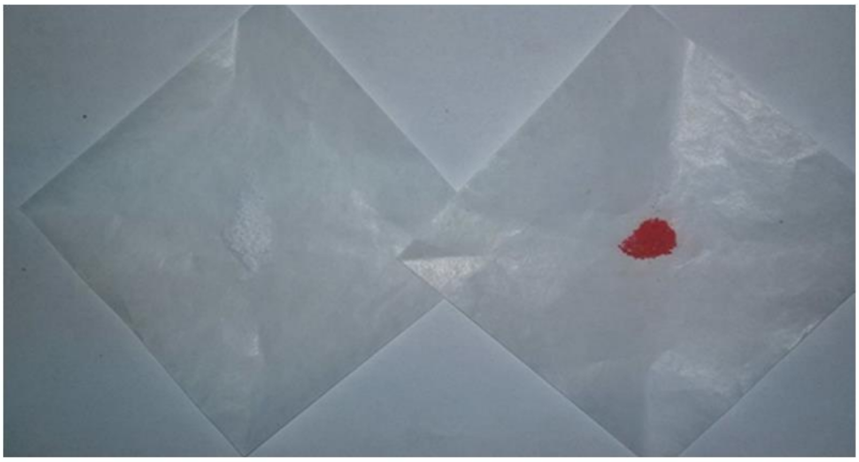

Figure S3 The visual appearance of MSN-CPN (left) and DOX/MSN-CPN (right).
